# PredictPTB: an interpretable preterm birth prediction model using attention-based recurrent neural networks

**DOI:** 10.1186/s13040-022-00289-8

**Published:** 2022-02-14

**Authors:** Rawan AlSaad, Qutaibah Malluhi, Sabri Boughorbel

**Affiliations:** 1 College of Engineering, Qatar University, Doha, Qatar; 2 Qatar Computing Research Institute, Hamad Bin Khalifa University, Doha, Qatar

**Keywords:** Deep learning, Predictive models, Attention mechanism, Electronic health record, Preterm birth, Pregnancy

## Abstract

**Background:**

Early identification of pregnant women at risk for preterm birth (PTB), a major cause of infant mortality and morbidity, has a significant potential to improve prenatal care. However, we lack effective predictive models which can accurately forecast PTB and complement these predictions with appropriate interpretations for clinicians. In this work, we introduce a clinical prediction model (PredictPTB) which combines variables (medical codes) readily accessible through electronic health record (EHR) to accurately predict the risk of preterm birth at 1, 3, 6, and 9 months prior to delivery.

**Methods:**

The architecture of PredictPTB employs recurrent neural networks (RNNs) to model the longitudinal patient’s EHR visits and exploits a single code-level attention mechanism to improve the predictive performance, while providing temporal code-level and visit-level explanations for the prediction results. We compare the performance of different combinations of prediction time-points, data modalities, and data windows. We also present a case-study of our model’s interpretability illustrating how clinicians can gain some transparency into the predictions.

**Results:**

Leveraging a large cohort of 222,436 deliveries, comprising a total of 27,100 unique clinical concepts, our model was able to predict preterm birth with an ROC-AUC of 0.82, 0.79, 0.78, and PR-AUC of 0.40, 0.31, 0.24, at 1, 3, and 6 months prior to delivery, respectively. Results also confirm that observational data modalities (such as diagnoses) are more predictive for preterm birth than interventional data modalities (e.g., medications and procedures).

**Conclusions:**

Our results demonstrate that PredictPTB can be utilized to achieve accurate and scalable predictions for preterm birth, complemented by explanations that directly highlight evidence in the patient’s EHR timeline.

## Introduction

Preterm birth (PTB) is defined as a delivery that occurs before the start of the 37th week of pregnancy, as opposed to full-term birth which occurs anytime from 37 to 42 weeks of gestation [1]. Worldwide, more than 15 million babies, or about 10−15*%* of all alive births, are born preterm every year [2, 3]. PTB accounts for over one third of infant mortality, and babies born preterm are at increased risk of significant long-term morbidity and disability, such as cerebral palsy, neurological disorders, behavioral problems, developmental delays, and mental health conditions [4–8]. Therefore, identifying pregnancies at risk for PTB and accordingly providing adequate interventions can improve both short- and long-term outcomes for babies born preterm.

The majority of existing work on PTB prediction aims to identify risk factors of PTB through a hypothesis-testing methodology, under highly-controlled settings. A number of risk factors have been reported to increase the risk of PTB such as: previous preterm labor, multiple gestation (being pregnant with more than one baby), diabetes, complications with the cervix, uterus, or placenta, tobacco smoking, and infections [9–11]. However, women who have preterm delivery often have no known risk factors [12]. In addition, some of the predictors (such as prior PTB) does not apply for first-time mothers. As a result, machine learning is of great interest to better predict PTB and several studies have attempted to predict PTB using machine learning techniques on a set of pre-defined clinical risk factors [13–20], or leveraging diverse variables from electronic health record (EHR) data [21]. More recently, few studies, with promising results, have used deep learning techniques to predict PTB using ultrasound and MRI images [22, 23], and high-dimensional EHR data [24, 25].

However, a majority of these studies reports poor to moderate predictive performance ranging from 59% to 75% ROC-AUC, ignores the sequential or temporal trajectory of events recorded in EHR, mostly used few human-derived features disregarding the huge amount of information embedded in each patient’s record, and rarely evaluated the model’s performance across multiple time points throughout the pregnancy timeline. To date, we lack effective predictive models for PTB, with two important challenges identified for deriving a PTB prediction model: (1) designing an accurate and scalable predictive model to handle the sequential high-dimensional EHR data, and being able to automatically select potential predictors from hundreds (if not thousands) of variables, and (2) complimenting these prediction models with reasonable interpretation mechanisms.

Attention mechanisms have been recently advocated to improve the accuracy as well as the interpretability of deep learning models. It was first introduced to improve the performance of the encoder-decoder recurrent neural networks (RNNs) on machine translation [26]. Recently, attention mechanisms have accomplished considerable success in many prediction tasks in healthcare [27–30]. Among these efforts, Choi et.al [27] proposed a model known as RETAIN, which uses a two-level neural attention model to predict heart failure using patient’s temporal EHR data. The predictive performance or RETAIN is comparable to recurrent neural networks (RNNs), while providing explanations for the visit-level and code-level contributions to the final prediction results.

In parallel, the expeditious growth in size and diversity of clinical data from electronic health records (EHR) has attracted the utilization of this data to predict a wide spectrum of clinical outcomes. However, EHR resources have been largely unexploited in the study of pregnancy. In contrast to other clinical contexts, the clinical surveillance of pregnancy data and its outcomes take place in a well-defined time frame, based on gestational length. Hence, EHRs seem to be very appropriate for modelling pregnancy complications, including preterm birth. To this end, predictive modeling using attention mechanisms with EHR data is anticipated to provide accurate individualized predictions for expecting mothers threatened with PTB.

In this work, we propose the PredictPTB model, an interpretable code-level attention-based recurrent neural network model to predict the risk of preterm delivery using multiple sources of temporal data from EHRs. We use a large dataset of 222,436 deliveries to demonstrate the predictive performance of the proposed model on the preterm birth prediction task compared to the original RETAIN model. We conduct a quantitative analysis to assess the effectiveness of the PredictPTB approach among several prediction points, data windows, and data modalities. Finally, we qualitatively examine the interpretability of the PredictPTB model by visualizing the learned attention-based weights and against the attention-based scores learned by the RETAIN model. The contributions of the paper can be summarized as follows:

- We propose a simplified version of the RETAIN architecture, where we employ RNNs to model the sequential patient’s EHR visits, and exploit a single code-level attention mechanism to improve the predictive performance while providing temporal code-level explanations for the prediction results.
- We compare the performance of our model across different combinations of data modalities, prediction points, and data windows to find an optimal combination for preterm birth prediction. We further compare these combinations of our model to other baseline models.
- We present a case-study of our model interpretability at visit-level and code-level, which illustrates how clinicians can gain some transparency into the predictions.

## Methods

### Problem formulation

Let *P*={*p*_1_,……*p*_*n*_} be a dataset of *n* patients. Each patient *p*_*j*_ EHR is comprised of a sequence of *T*_*j*_ patient visits, $p_{j}=\{x_{1}, x_{2}, \dots, x_{T_{j}}\}$, ordered by visit date *t*∈{1,*T*_*j*_}, where the last time point *T*_*j*_ denotes the time on which the delivery for patient *p*_*j*_ has occurred. We express medical events in EHR as medical codes (e.g. diagnoses, medications, procedures, and lab orders), denoted as {*c*_1_,*c*_2_,…,*c*_|*C*|_}∈*C*, where *C* represents the entire set of unique medical codes. Each visit *x*_*i*_ can be expressed as a binary vector *x*_*i*_∈{0,1}^|*C*|^, where the *k*-th element is set to 1 if the *i*-th visit contains the medical code *c*_*k*_, otherwise it is set to 0. Let *m*={*m*_1_,…,*m*_*p*_} be the set of prediction time points. Given the EHR history *p*_*j*_={*x*_1_,*x*_2_,…,*x*_*T*−*m*_} of each *j*^*th*^ patient up to time point *T*−*m*, our task is to predict the risk of a PTB at time point *T*, denoted as $\hat {y}_{T}^{j} \in \{0,1\}$, and accurately interpret why a patient is predicted as PTB, using the patient’s temporal EHR history. To address this problem, we introduce a code-level attention-based RNNs to provide an interpretable clinical risk prediction model for preterm birth.

### Preliminaries on attention mechanism

**Fig. 1:**
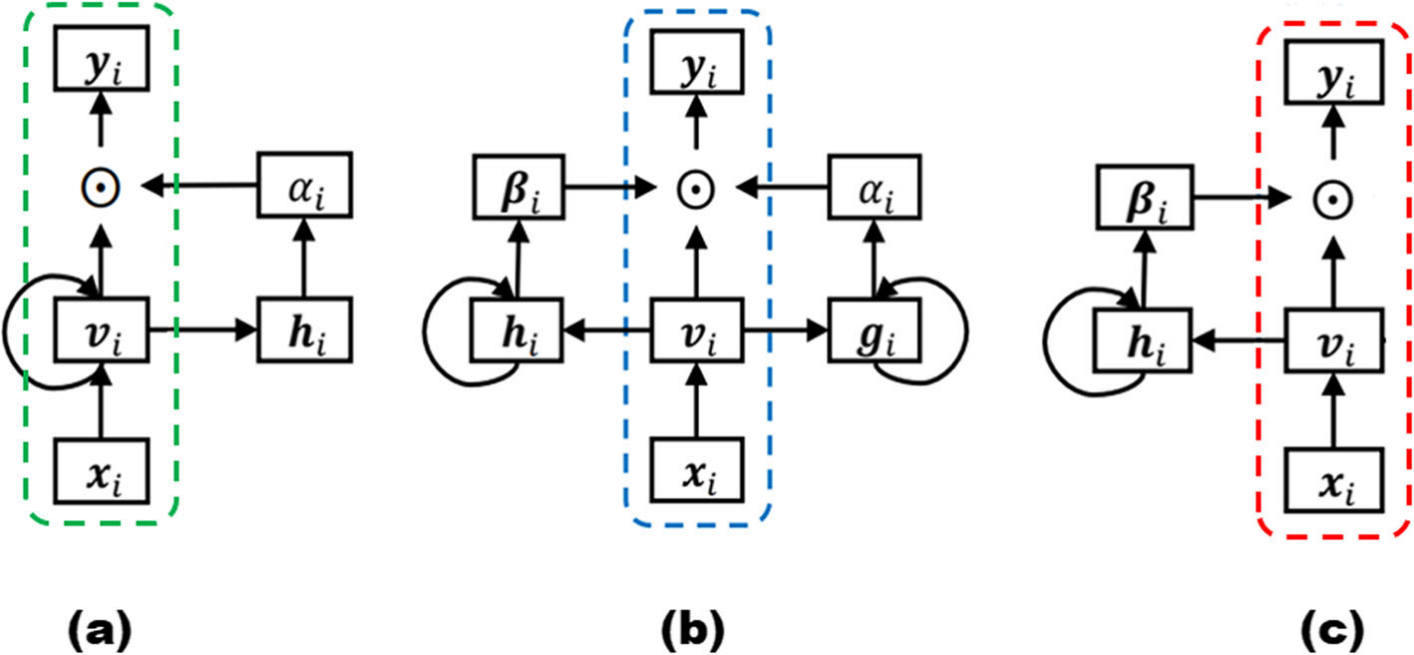
**A** Standard attention model, **B** RETAIN model, **C** PredictPTB model

Attention mechanism has been an important component in recurrent neural networks (RNNs) to capture long-term dependencies. It computes the dynamic weights representing the relative importance of the inputs in a sequence for a particular output. Figure 1(a) illustrates the architecture of a standard attention model, in which the attention mechanism summarizes the source sequence information in the encoder RNN hidden states (i.e., *h*_*i*_), computes the dynamic attention scores for each visit *v*_*i*_ as *α*_*i*_, and then multiplies the weights *α*_*i*_ with the input sequence *v*_*i*_ to weight the sequence. A single context vector *c*_*i*_ for a patient up to the *i*-th visit can then be calculated using the sum of the weighted vectors as:

1 $$ c_{i}= \sum\limits_{j=1}^{i}\alpha_{j} \odot v_{j}  $$

### Reversed time attention model (RETAIN)

The RETAIN model was first introduced in [27] for the prediction of heart failure using patient’s longitudinal EHR data. Given patient records, RETAIN can make accurate predictions, comparable to RNNs, while explaining how each medical code at each visit contributes positively or negatively to the final prediction score. RETAIN is based on a double-attention mechanism, which integrates two single-attention models (the visit-level attention RNN _*α*_ and the code-level attention RNN _*β*_) to generate the patient representation, as illustrated in Fig. 1(b). Using the computed attention weights at visit-level *α* and code-level *β*, the context vector *c*_*i*_ for a patient up to the *i*-th visit is calculated as:

2 $$ c_{i}= \sum\limits_{j=1}^{i}\alpha_{j} \beta_{j} \odot v_{j}  $$

### Architecture of the PredictPTB model

The RETAIN architecture seems to have a redundant attention branch for capturing visit-level attentions, which are inherently available in the code-level attentions. Therefore, to construct a more precise contextual representation of each patient, we introduce the PredictPTB model. Our model simplifies the RETAIN architecture into a single code-level attention layer RNN _*β*_. This approach reduces the complexity of the RETAIN architecture while improving the accuracy of the predictions due to 1) directly promoting the code-level information in each step of the model, 2) paying more attention to representative and discriminative features than other features, and 3) limiting the number of model parameters which reduces the risk of over-fitting and possible gradient flow. We use bidirectional RNNs, specifically BiLSTMs, which enable both future and past information to be accessible by the current state, providing more information about the input. This mimics the practice of a clinician examining a patient’s EHR both forward and backward, trying to identify a set of weights representing the relative importance of patient’s individual visits or codes within those visits.

**Fig. 2:**
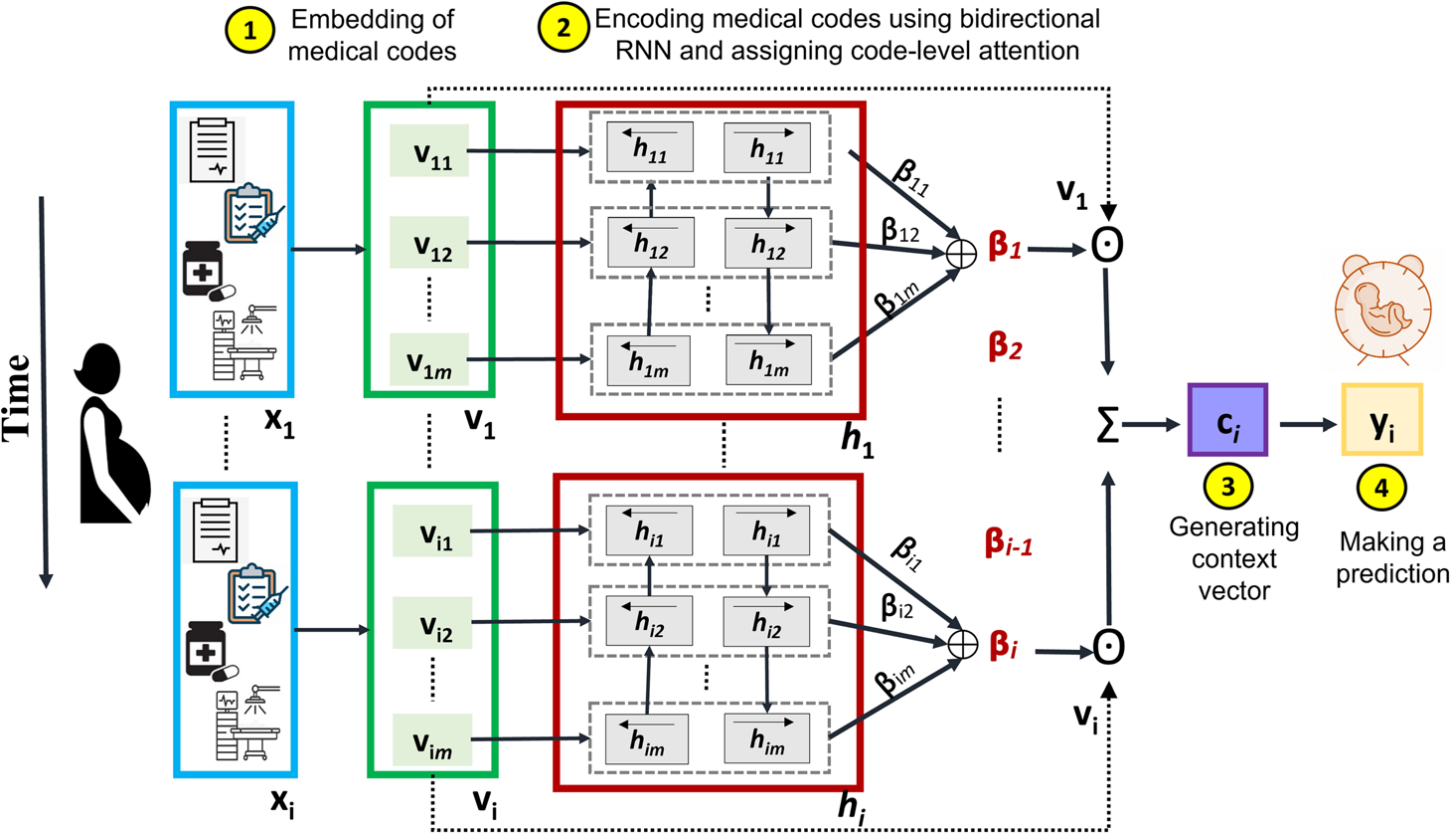
An overview architecture of our code-level attention model (PredictPTB)
  where $v_{i} \in \mathbb {R}^{m}$ is the embedding of $x_{i} \in \mathbb {R}^{C}, W_{emb} \in {\mathbb {R}}^{m \times C}$ is the embedding matrix, *m* is the embedding size across *C* medical variables, *σ* is a non-linear activation function such as rectified linear unit (ReLU) or sigmoid, and *b*_*x*_ is the bias.
- **Step 2**: The embeddings are fed as inputs to a recurrent neural network RNN _*β*_, which computes the attention-based contribution scores of individual medical variables and generate code-level attention weights *β*_*i*_. We highlight here that there are two main differences between standard RNN with attention architecture shown in Fig. 1(a) and PredictPTB architecture illustrated in Fig. 1(c). First, in standard RNN with attention, the recurrence is on the hidden state vector *v*_*i*_, which hinders the interpretation of the model. On the other hand, in PredictPTB, the recurrence is on the attention generation component *h*_*i*_ while the hidden state *v*_*i*_ is generated by a simpler more interpretable output. Second, in standard RNN with attention, the scalars *α*_1_,…,*α*_*i*_ are the visit-level attention weights that control the contribution of each visit embedding *v*_1_,…,*v*_*i*_. In PredictPTB, The vectors *β*_1_,…,*β*_*i*_ are the code-level attention weights that focus on each coordinate of the visit embedding *v*_1,1_,*v*_1,2_,…,*v*_1,*m*_,…,*v*_*i*,1_,*v*_*i*,2_,…,*v*_*i*,*m*_.
  Also, in contrast to the RETAIN architecture shown in Fig. 1(b), PredictPTB eliminates the RNN _*α*_ layer and use a single attention layer RNN _*β*_ to generate the weights, as follows:
  4 $$ \begin{aligned} h_{i}, h_{i-1}, \dots, h_{1} = RNN_{\beta}(v_{i}, v_{i-1},\dots, v_{1}) \\ \beta_{j}= tanh(W_{\beta}h_{j} + b_{\beta}) \; \; \; \; \text{for} \; j=1,\dots,i. \end{aligned}  $$
  where $h_{i} \in \mathbb {R}^{q}$ is the hidden layer of RNN _*β*_ at time step *i*, *q* is the hidden size of RNN _*β*_,*β*_*j*_ is the attention weight for individual variables, $W_{\beta } \in \mathbb {R}^{m \times q}$ and $b_{\beta } \in \mathbb {R}^{m}$ are parameters to learn.
- **Step 3**: The computed attention weights are used to generate the patient representation context vector *c*_*i*_ as:
  5 $$ c_{i}= \sum\limits_{j=1}^{i} \beta_{j} \odot v_{j}  $$
  where $c_{i} \in \mathbb {R}^{m}$
- **Step 4**: The predictions of our model can then be computed by linearly transforming the context vectors *c*_*i*_ using:
  6 $$ \hat{y_{i}}= Softmax (W c_{i} + b)  $$
  where $W \in \mathbb {R}^{m}$ and $b \in \mathbb {R}$ are the parameters to learn.

The predictions of our proposed PredictPTB model are made using the steps described in Fig. 2, as follows:

- **Step 1**: The model embeds a patient’s visit sequence *v*_*i*_ as:
  3 $$ v_{i}= \sigma(W_{emb} x_{i} + b_{x})  $$

### Interpreting PredictPTB model

To interpret predictions made by our PredictPTB model, we follow the interpretation approach described in [27]. Given a patient’s list of visits *x*_1_,…,*x*_*i*_, the probability of the binary output vector *y*_*i*_∈{0,1} can be predicted as follows:

7 $$ \mathbf{p}\left(y_{i}\mid x_{1}, \dots,x_{i} \right)= \mathbf{p}(y_{i}\mid c_{i})= Softmax \left(W \left(\sum_{j=1}^{i} \beta_{j} \odot v_{j}\right) + b\right)  $$

8 $$ \mathbf{p}(y_{i}\mid x_{1}, \dots,x_{i})= Softmax \left(W \left(\sum_{j=1}^{i} \beta_{j} \odot \sum_{k=1}^{C} x_{j,k} W_{emb} [:,k]\right) + b\right)  $$

9 $$ = Softmax \left(\sum_{j=1}^{i} \sum_{k=1}^{r} x_{j,k} W \left(\beta_{j} \odot W_{emb} [:,k]\right) + b\right)  $$

where *x*_*j*,*k*_ is the *k*-th element of the input visit *x*_*j*_. In order to compute the contribution *ω* of the *k*-th code at each visit *x*_*j*_ at time step *j*≤*i* for predicting *y*_*i*_, we deconstruct Eqs. 9 into 10, where we exclude the index *i* of *y*_*i*_ in the *β*_*j*_, as follows:

10 $$ \omega (y_{i},x_{j,k})= \underbrace {W \left(\beta_{j} \odot W_{emb} [:,k]\right)}_{\text{Contribution coefficient}} \underbrace{x_{j,k}}_{\text{Patient visit}}  $$

In the real clinical practice, clinicians typically identify different weights on different visits and medical codes, as part of the diagnosis process. In this sense, the above contribution coefficient can be used to highlight important visits and medical codes.

### Preterm birth: data modalities, prediction points, and data windows

#### Data modalities

For each patient, we extracted data from multiple domains of sources in EHR to enrich the patient representation with multiple data modalities. The modalities include diagnosis, medications, procedures, and lab orders. The data of each modality was represented as a concept in a set of standardized terminologies, including ICD10 and ICD9 for diagnosis, NDC and brand names for medications, CPT and ICD9 for procedures, and LOINC for lab orders. The numbers of unique medical concepts representing diagnosis, medications, procedures, and lab orders were 14795, 1332, 7640, and 3333, respectively.

#### Prediction points

To further elaborate on the advantages of using the PredictPTB model, we design experiments to quantify the performance across different prediction time points during the pregnancy timeline. In similar studies which develop predictive models for preterm delivery using EHR data, the gestational age is learned from a combination of ICD codes, gestational age extracted from discharge summaries, and gestational age calculated via EDD (estimated due date). However, our dataset does not contain enough information about the gestational ages of the pregnancies and the timestamps for the start of the pregnancies are unknown. Therefore, we could not use the start of the pregnancy as the reference point in our prediction setup. Instead, we used the ICD codes to ascertain the actual delivery date and used it as our reference point. Our setup may provide some advantages compared to using the gestational age as the prediction reference. For example, the accuracy of the gestational age calculation, which is also used to calculate the estimated date of delivery, is already a debatable subject in the obstetric community [31, 32]. Previous literature reported that only 4−5*%* of women deliver on their due date. Therefore, having an additional perspective for the same pregnancy timeline using the actual delivery date as a reference instead of the gestational age, which is only an estimate, might be able to provide more accurate predictions for clinicians. Moreover, a previous work on preterm prediction using EHR data [25] evaluated the prediction performance using the gestational age as a reference point compared to the prediction performance when the reference point is the delivery date. The paper reported that both setups yielded similar results.

**Fig. 3:**
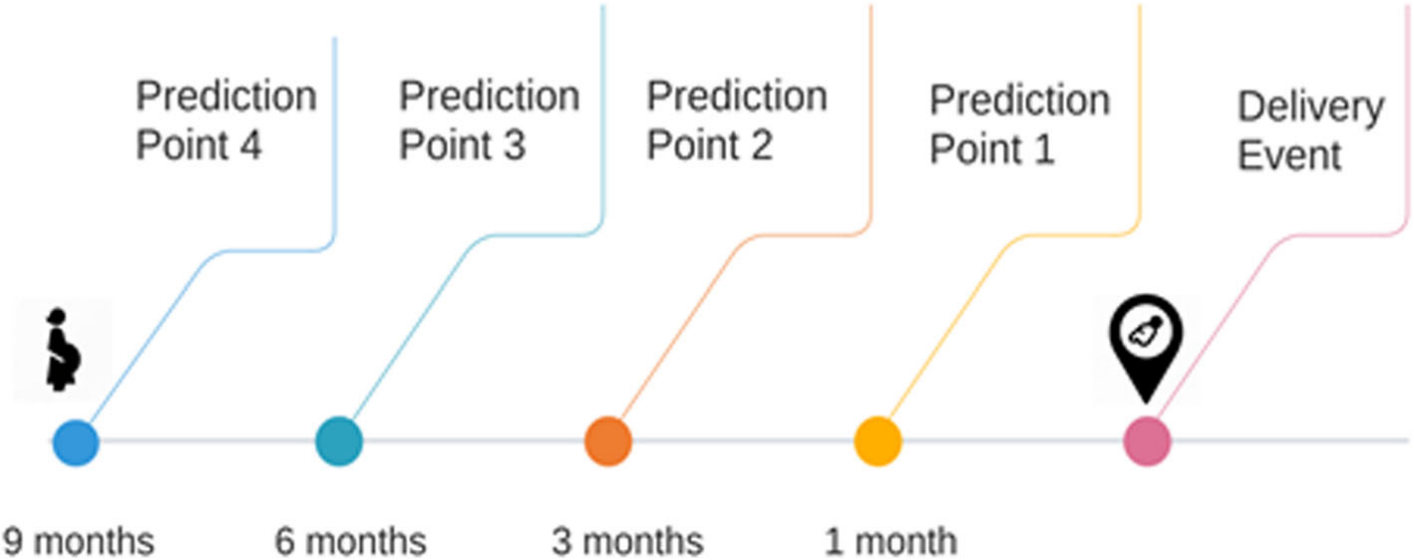
Prediction points used in the analysis

In this work, we have used the delivery event as our reference point for predicting the risk of preterm birth at 1, 3, 6, 9 months, before the delivery event. We refer to these prediction points as P1, P2, P3, and P4, as shown in Fig. 3. For each prediction point, the patient EHR history up to the prediction point is used for the prediction. For example, if the prediction point is 3 months before delivery, only the EHR data up to the prediction point is used by the model, and the data between the prediction point and the delivery event is discarded. This represents real clinical scenarios, where the physician needs to predict the risk of preterm delivery at different time points of the pregnancy timeline.

#### Data windows

We used two setups for EHR data windows; long-term (full history) data window and short-term (pregnancy history) data window. In the long-term window setup, all EHR data of the patient up to the prediction point X is used to train the predictive models. In the short-term window setup, only the EHR data between the start of the pregnancy up to the prediction point X is used, and previous history before pregnancy is discarded. These two data windows will help us assess the influence of the events happening during the pregnancy timeline on the predictive performance of the model, compared to the influence of all events included in the patient EHR history before and after the start of the pregnancy.

## Experiments and results

### Dataset

To validate the predictive performance of the proposed model, we conducted experiments on a retrospective and longitudinal cohort of full-term and preterm deliveries obtained from the Cerner Health Facts database. The extracted dataset includes more than 222,000 deliveries from about 300 healthcare centers in the United States between 2000-2017. Gestational ages of the pregnancies were not available in the data. Therefore, we relied on ICD-9 diagnosis codes to identify preterm and full-term pregnancies and labeled the data accordingly. Preterm deliveries were identified using ICD-9 codes under 644.2x. Full-term deliveries were identified using ICD-9 codes 645.xx, 649.8, 650 and 652.5. Tables 1 and 2 describe the statistics of the two cohort setups: long-term window and short-term window, respectively.

**Table 1:** Summary statistics for long-term window cohort

Cohort: Full History	Total #pregnancies	Counts for each class	Mean age	Average #visits
**P1 (1 month)**	222436	Fullterm 204700	28.03	12.19
		Preterm 17736	28.76	13.37
**P2 (3 months)**	202930	Fullterm 187073	27.81	10.96
		Preterm 15857	28.42	12.26
**P3 (6 months)**	177253	Fullterm 163641	27.45	10.1
		Preterm 13612	27.84	11.51
**P4 (9 months)**	150904	Fullterm 138468	26.91	9.92
		Preterm 12436	27.43	11.24

**Table 2:** Summary statistics for short-term window cohort

Cohort Pregnancy History	Total #pregnancies	Counts for each class	Mean age	Average #visits
**P1 (1 month)**	168932	Fullterm 155647	28.15	6.96
		Preterm 13285	29.08	7.07
**P2 (3 months)**	133704	Fullterm 123968	28.04	5.16
		Preterm 9736	28.87	5.27
**P3 (6 months)**	77779	Fullterm 73477	27.95	3.33
		Preterm 4302	28.56	3.28

The medical record of each patient encompasses the following data modalities: diagnoses, procedures, medications, and lab orders. We use a statistical classification system, presented by several types of coding systems specific to data modalities, to represent the features of each patient in the dataset. Diagnostic codes are used to describe diseases, disorders and symptoms. We use the International Statistical Classification of Diseases and Related Health Problems (known as ICD) for diagnosis codes, specifically, the ICD-9-CM and ICD-10-CM systems. Procedural codes are numbers or alphanumeric codes used to identify specific health interventions taken by medical professionals. We use two coding systems for procedural codes: the Current Procedural Terminology (CPT) and ICD-10-PCS (Procedure Classification System). Pharmaceutical codes are used to identify medications. We use the National Drug Code (NDC) system, which is a unique 10-digit or 11-digit, 3-segment number, used as a universal product identifier for human drugs. For laboratory test orders and results, we use Logical Observation Identifiers Names and Codes (LOINC).

The temporal models used in this work requires patient-level time-ordered data that has been collected over time. Therefore, we chose to present our EHR dataset in the form of list of lists of lists. The outermost list corresponds to patients, the intermediate list corresponds to the time-ordered visit sequence each patient made, and the innermost list corresponds to the medical codes that were documented within each visit. Furthermore, to ensure that the RNN models have sufficient number of visits to train on, only patients who have at least two visits in their EHR were included.

### Baselines

Our PredictPTB model was evaluated against two baselines: the Multi-Layer Perceptron (MLP) and the RETAIN models. For the MLP model, we combine features extracted from all the visits for a patient into a single feature vector. To do this, we use the counts for each medical code in the patient’s list of visits. The resulting vector was used to train an MLP model.

### Experimental Setup

Models were trained on a DGX-1 server equipped with 8 NVIDIA Tesla V100 GPU accelerators. Models were trained for the task of predicting whether the expecting mother will deliver a full-term or a preterm baby. We implemented PredictPTB using Tensorflow 2.0+ framework. For all models, patients were randomly split into training (70*%*), validation (10*%*), and test (20*%*) sets. The same proportion of preterm and full-term deliveries was maintained among the training, validation, and test sets. The following parameter values were used for the PredictPTB and RETAIN models: embedding size= 200, RNN hidden size= 200, batch size= 32. To help conserve GPU RAM, we set the maximum number of visits for a patient to 200 visits. For patients with more than 200 visits, the most recent 200 visits will be used. The performance of the models is reported on the test set, and the following evaluation metrics were used: ROC-AUC (Area Under the Receiver Operating Characteristic Curve), PR-AUC (Area Under the Precision-Recall Curve), sensitivity, and specificity. The PR-AUC curves are well-suited for imbalanced settings, where the focus of the PR curve on the minority class makes it an effective diagnostic for imbalanced binary classification models [33].

### Results

We present the results of preterm birth prediction on the EHR dataset by both the baselines and our PredictPTB model. All models were trained at four prediction time points 1 month (P1), 3 months (P2), 6 months (P3), and 9 months (P4) before the delivery, and on two data history setups: long-term and short-term windows.

#### Prediction performance across prediction time points and data windows

**Fig. 4:**
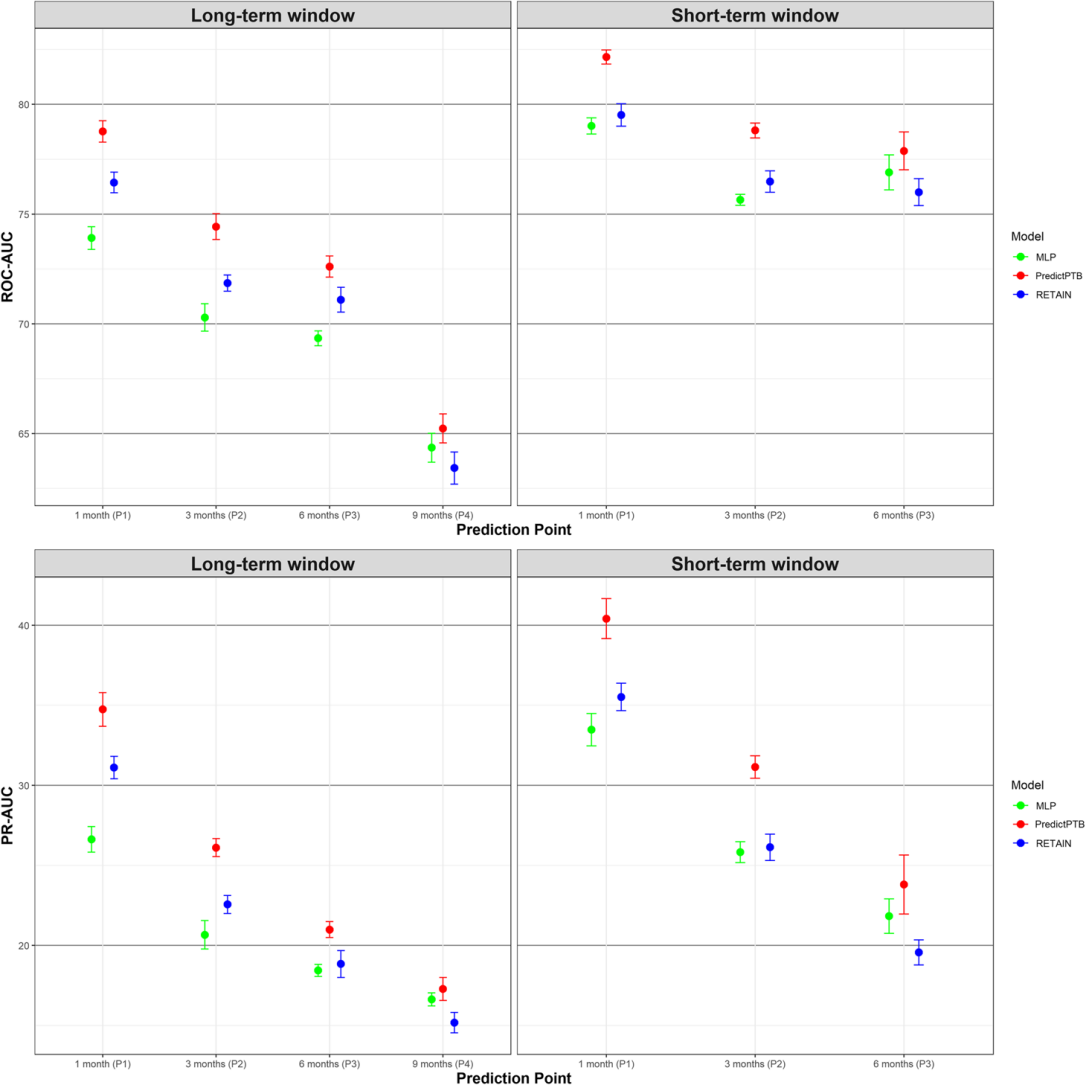
Predictive performance of the implemented models across the four prediction points using all data modalities for long-term and short-term data windows

The objective of this experiment is to compare how models trained using combined data modalities perform in different scenarios, represented by four prediction time points and two data history setups. Figure 4 shows that our PredictPTB model consistently provides more accurate predictions, as compared to the RETAIN and MLP models, across the four prediction points. Models achieve the best performance at 1 month before delivery, using the short-term window history, where our PredictPTB model performed better than both baselines. For this best performance scenario, PredictPTB achieved a PR-AUC and ROC-AUC of 40.4*%* and 82.2*%*, compared to 35.5*%* and 79.5*%* for RETAIN, and 33.5*%* and 79.0*%* for MLP. In addition, results show that models improved in performance as the prediction point got closer to the delivery date. For example, using the long-term window setup, our PredictPTB model was able to predict the risk of preterm birth at P1 (1 month before delivery) with a PR-AUC and ROC-AUC of 34.7*%* and 78.8*%*, compared to 26.1*%* and 74.4*%* at P2 (3 months), 21.0*%* and 72.6*%* at P3 (6 months), and 17.3*%* and 65.2*%* at P4 (9 months). Moreover, the proposed PredictPTB uses a single attention mechanism to capture both visit and code-level contribution. Therefore the model size is smaller compared to RETAIN and less prone to over-fitting.

Moreover, our analysis confirm that EHR features collected after the start of the pregnancy can better identify preterm births, compared to combining features collected before and after the start of the pregnancy. For example, using the short-term data window, PredictPTB achieved a PR-AUC of 40.4*%*, compared to 34.7*%* for long-term window at P1. This finding confirms the general intuition that the short-term condition of a patient is usually the most determinant of health outcome.

#### Prediction performance using single data modality vs. combined data modalities

**Fig. 5:**
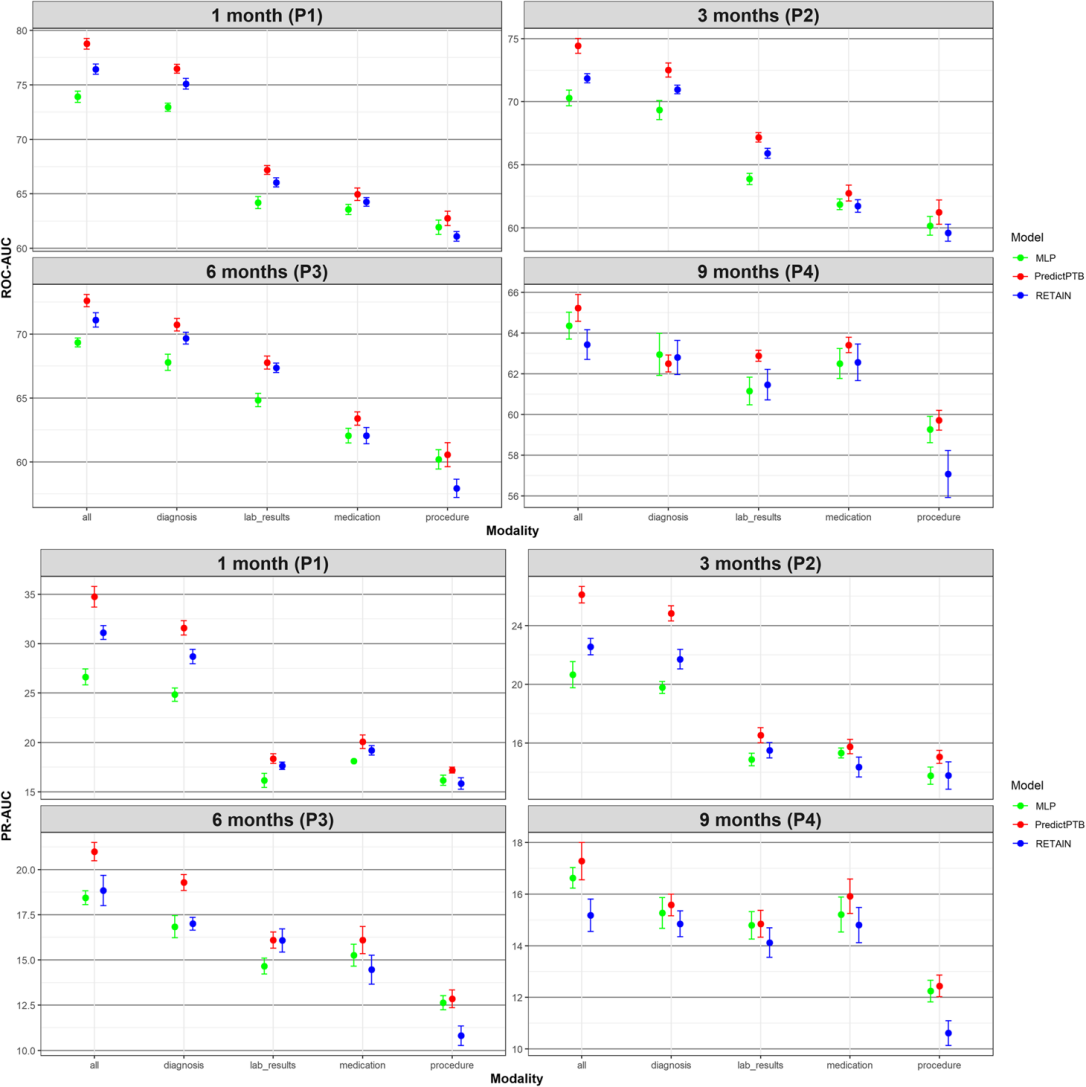
Predictive performance of models trained on single modality and integrated modalities of data, across the four prediction points using long-term data window

**Fig. 6:**
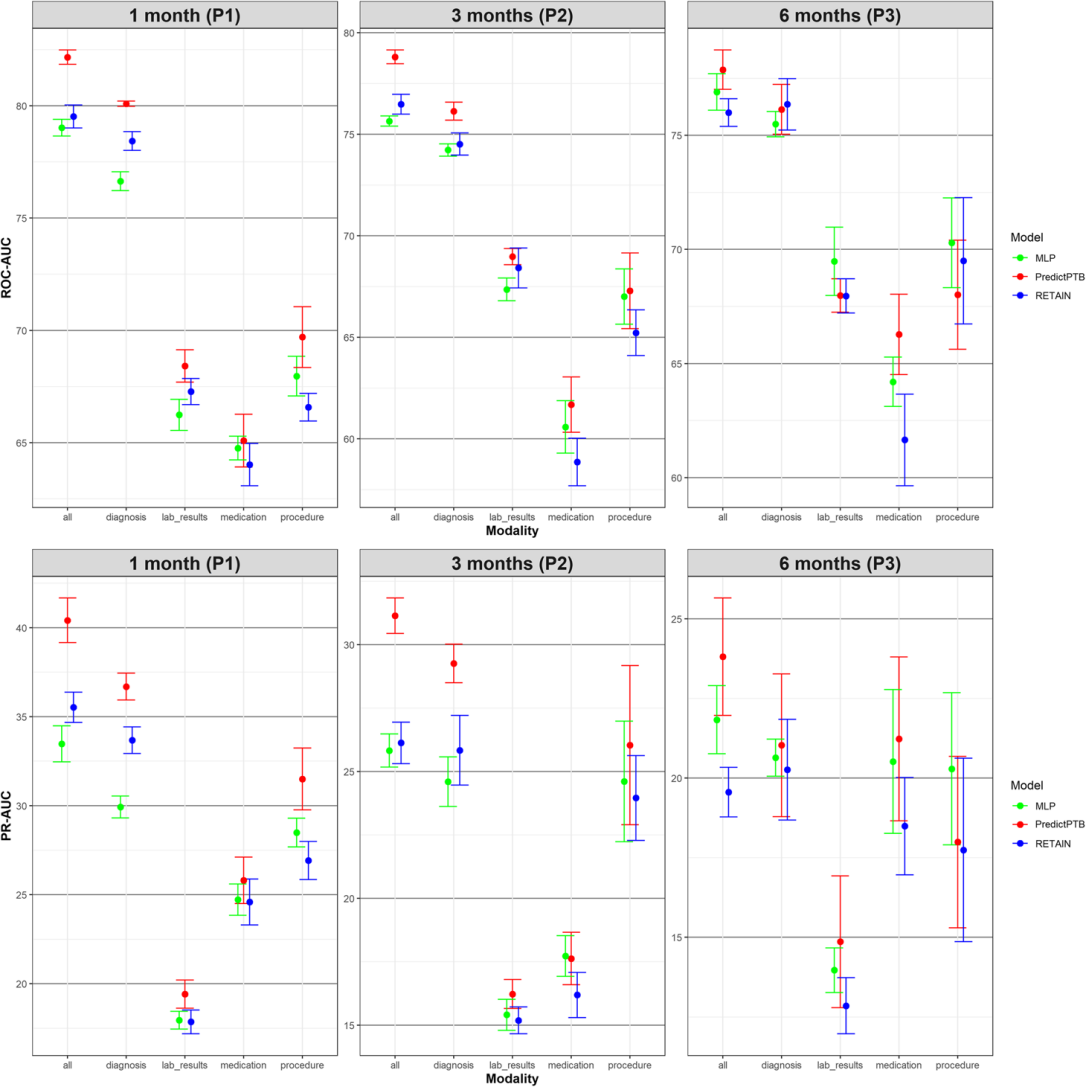
Predictive performance of models trained on single modality and integrated modalities of data, across the three prediction points using short-term data window

The objective of this experiment is to evaluate the prediction performance of models trained on each individual data modality compared to models trained on all modalities combined together. Results presented in Figs. 5 and 6 show that combining all modalities together provides the best predictive performance, compared to using individual modalities. As for individual data modalities, diagnosis data achieved the highest performance across all prediction points and data windows. Diagnosis codes represents the most condensed information about patient status and history. While the main purpose of these codes are for billing purposes, it has been widely shown that they are highly predictive of patient health outcome [34]. At P1, diagnosis data was able to predict preterm delivery with PR-AUC and ROC-AUC of 36.7*%* and 80.1*%* for short-term window, and 31.6*%* and 76.5*%* for long-term window. As for data modalities, results demonstrated that combining all modalities together (diagnosis, medication, procedures, and lab results) improved the PR-AUC by 10% compared to using the diagnosis modality alone. The performance of other modalities varied among different data windows and prediction points. For example, procedures data was the least predictive modality for long-term window across all prediction time points. For these results, we note that observational modalities (diagnosis and lab orders) are more predictive than intervention modalities (medication and procedure). Since interventions can be subject to doctor opinion and understanding of the patient history and condition, it might explain the reason why it is less predictive than the observational data. Another explanation is that the data frequency for interventional data in EHR is lower than observational data. Hence model training is more reliable based on diagnosis and lab order modalities. On the other hand, for short-term window, procedures modality performed better than medications for the three prediction points P1, P2, and P3, and better than lab results for P1 and P3. This highlights that procedures performed after the start of the pregnancy (short-term window), are better predictors for preterm birth, compared to medications and lab results ordered during pregnancy.

### Model interpretation for preterm birth prediction

We present a use case to demonstrate the ability of our PredictPTB model to explain individual prediction results, at visit-level and code-level, for preterm birth prediction. In addition, we compare the visit-level contributions generated by the PredictPTB and RETAIN models. For PredictPTB, code-level contributions are computed as described in Eq. 10, and visit-level contributions are computed by aggregating the contributions of individual codes included within a visit.

#### Code-level and visit-level interpretations

**Fig. 7:**
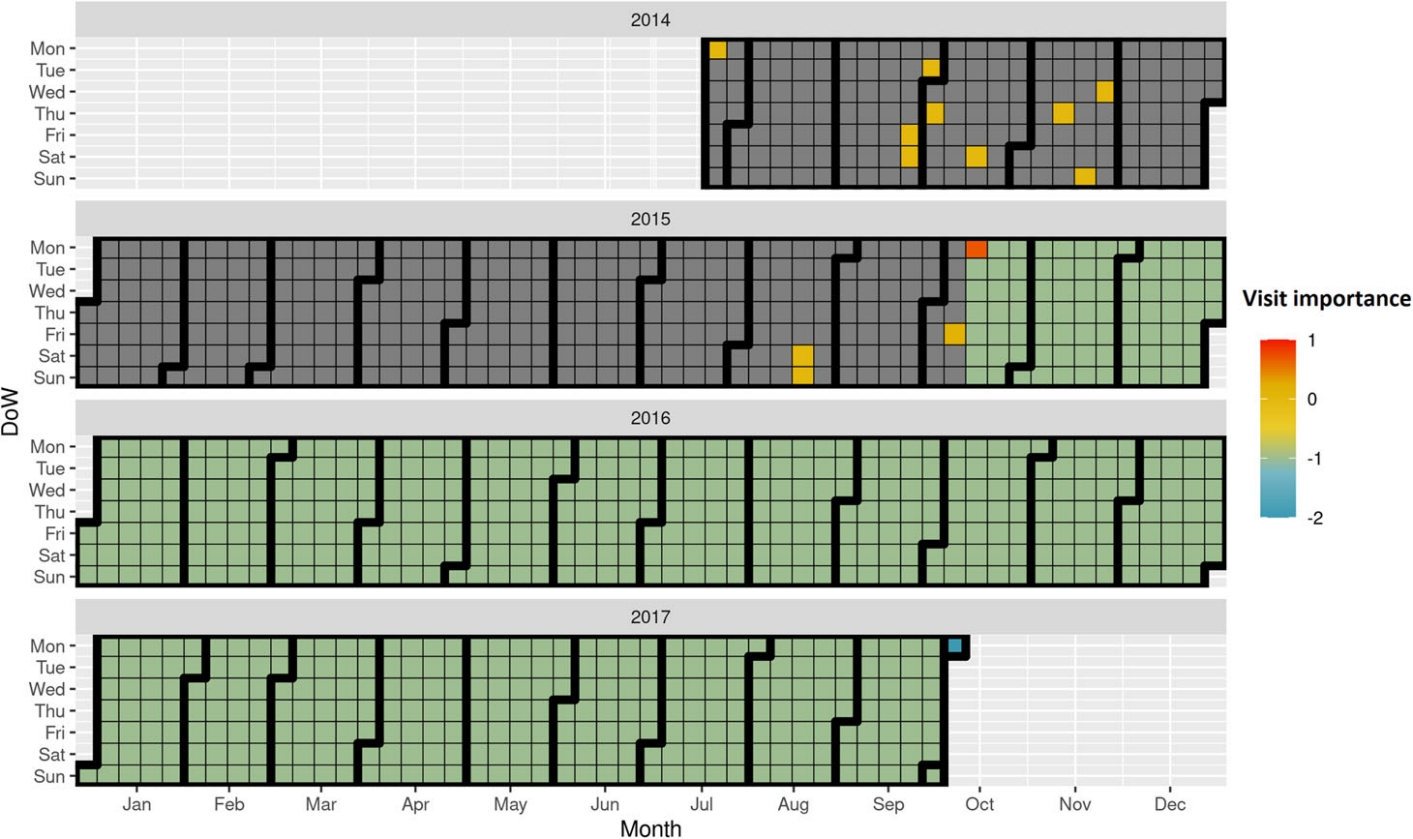
Temporal visualization of visit-level contributions over a patient’s EHR timeline, using PredictPTB model trained to predict preterm birth

**Fig. 8:**
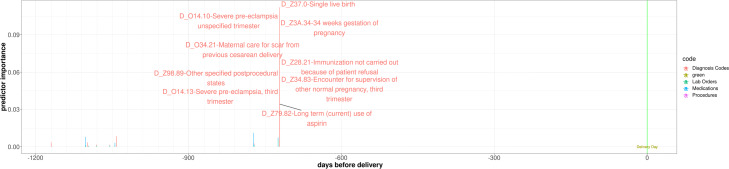
Interpretation of prediction results over a patient’s EHR timeline. The code-level attribution in each visit is shown along the x-axis (i.e. time) with the y-axis representing the magnitude of individual codes contributions to preterm birth in each visit

In Fig. 7, we show an example for a patient from the test set, predicted as preterm delivery using the PredictPTB model. The blue square (in Oct 2017) indicates the delivery date. The attention weights show strong contribution of a previous visit, about two years prior to delivery. Looking closer at the medical codes included in this visit (Fig. 8), we can see that a previous single live birth delivery at 30-34 weeks of gestation (preterm) occurred on that day, due to severe preeclampsia. These findings are in line with published literature reporting that a history of prior preterm birth and preeclampsia in a previous pregnancy are major risk factors for preterm delivery in subsequent pregnancies [35, 36]. In addition, the codes included in this visit indicate a long-term and current use of aspirin during the previous pregnancy, probably to prevent preterm preeclampsia. Moreover, the codes on this visit report that this patient had a previous cesarean delivery, which might also increase the risk of preterm birth in later pregnancies [37, 38]. This patient is a great example demonstrating the ability of the attention mechanism to go beyond recent events and model long-range dependencies among medical events to the predicted outcome.

#### Comparison of visit-level attributions of PredictPTB and RETAIN models

**Fig. 9:**
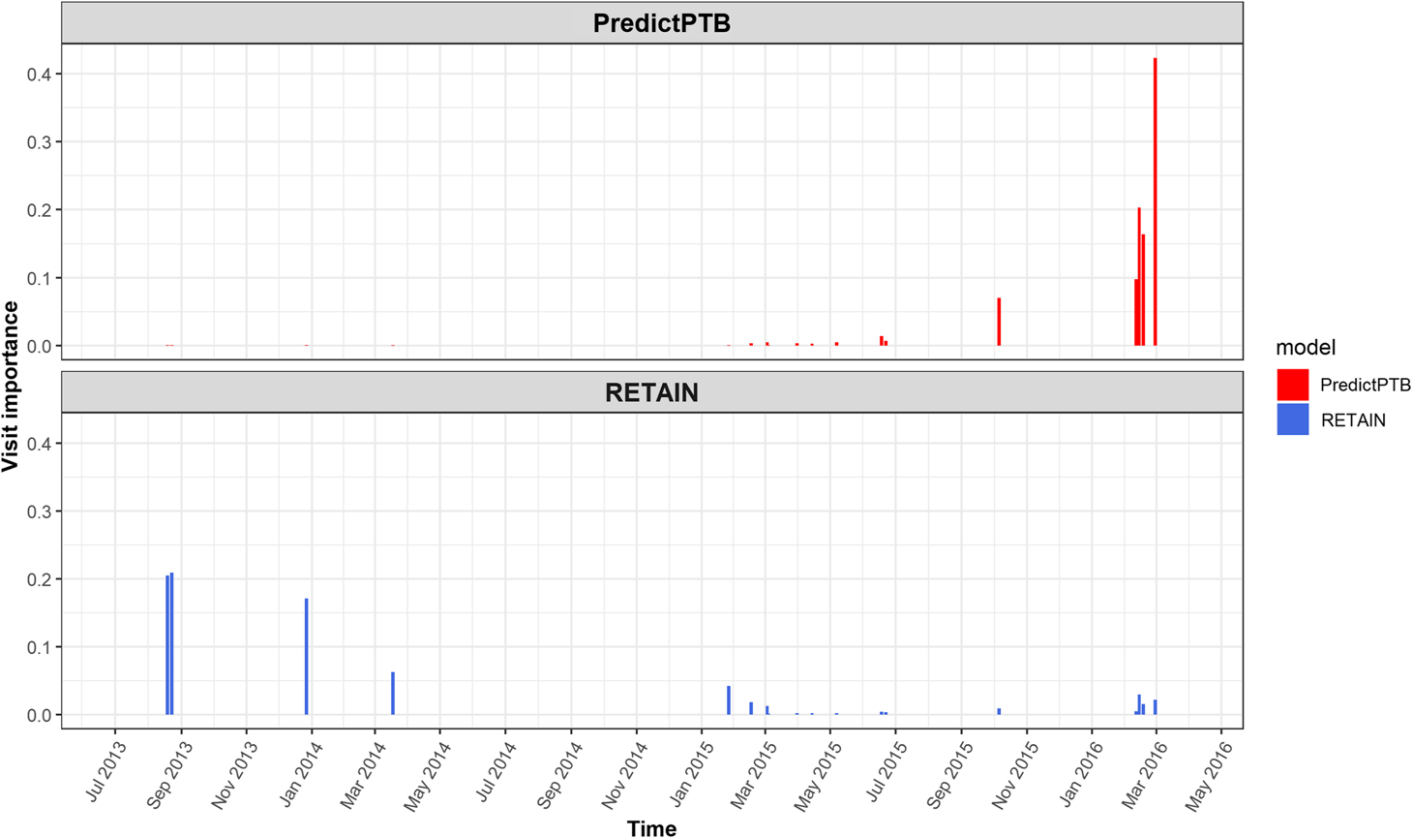
Comparison of visit-level attributions between PredictPTB and RETAIN models

Here, we show an example for a use-case, where PredictPTB was able to produce more clinically-relevant explanations for preterm prediction than RETAIN, using data available up to one month before the delivery event. In Fig. 9, we can observe that PredictPTB highlights a few visits, a month before the delivery, with high contributions in February 2016, while RETAIN highlights some older visits with high contribution between August 2013 and December 2014. The visits which were highlighted by the PredictPTB model includes the following codes: F41.9: Anxiety disorder, J02.9: Acute pharyngitis, J06.9: Acute upper respiratory infection, and 18481-2: Culture Throat and Group A Beta Strep AG Rapid Screen Qualitative, while visits highlighted by the RETAIN model includes the these codes: Z32.01: Encounter for pregnancy test result positive, Z30.9: Encounter for contraceptive management, and N76.0: Acute vaginitis. For this patient, PredictPTB visit attributions seem to be more clinically-relevant than RETAIN attributions, since having an acute upper respiratory infection such as acute pharyngitis was found to be positively correlated with preterm birth [39, 40]. In addition, anxiety has been reported in several studies as a risk factor for preterm birth [41, 42]. The visits highlighted by the RETAIN model, are about three years prior to the delivery, which makes them less relevant, especially not reporting potential risk factors for preterm delivery, except for acute vaginitis, which might have only affected the previous pregnancy and not the current one [43]. There is currently no literature reporting that acute vaginitis in a pregnancy increases the risk of preterm birth in subsequent pregnancies.

This use-case demonstrates the ability of the PredictPTB model to utilize the computed contributions of individual medical codes to explain code-level and visit-level contributions to the model’s prediction for a particular patient. In addition, PredictPTB may be able to provide more precise interpretations than RETAIN for preterm birth predictions. The use of a single attention layer for both code-level and visit-level attributions seems to provide a more consistent interpretations compared to two separate attention layers as in RETAIN.

## Discussion

We introduced the PredictPTB model to predict the risk of preterm birth, using patient EHR longitudinal data. The core component of our model is a code-level attention-based RNN, which can embed relevant contextual information into each medical code to generate code-level attention weights. We demonstrated the prediction performance of our model on the task of predicting the risk of preterm birth, up to 9 months earlier than its occurrence, using data routinely collected in EHR.

Results showed that our PredictPTB model performed better than the RETAIN model, across all prediction points, data windows, and data modalities. The PredictPTB model achieved the best predictive performance at 30 days before delivery, with an ROC-AUC of 82.2*%* and PR-AUC of 40.4*%*, when using combined data modalities and pregnancy history setup. As for data modalities, results demonstrated that combining all modalities together (diagnosis, medication, procedures, and lab results) improved the PR-AUC by 10% compared to using the diagnosis modality alone and improved the ROC-AUC for about 4%. This suggests that combination of all data modalities have the potential to quantify PTB risk better than using the diagnosis data alone, due to enrichment of individual patient representation with multiple data sources. Moreover, results showed that the use of models trained on data collected after the start of the pregnancy improved the performance by up to 5% on ROC-AUC, compared to models trained on the full patient EHR history, which includes both data before and after the start of the pregnancy. This supports previous findings in [44–46], which indicates that the most important risk factors are associated with events happening during the pregnancy timeline.

The PredictPTB model has a number of architectural advantages over previous methods for modelling EHR data for prediction of clinical outcomes. Firstly, the attention-based approach we implemented is more interpretable than other black-box deep learning methods, which often lack the capability of identifying features driving predictions. This property enables clinician to better understand risk factors associated with the predicted clinical outcomes. Second, our flexible architecture enables capturing additional modalities of EHR data (e.g. surgeries, clinical notes, etc.), by simply adding a fifth or sixth (or more) list of concepts to our embedding layer. Third, PredictPTB is based on a relatively simple architecture compared to previous methods such as RETAIN, and GRAM [28]. PredictPTB reduces the complexity of the RETAIN architecture while improving the accuracy of the predictions. Simple models are known to provide three main advantages over complex models: prevent overfitting, improve interpretability, and increase computational efficiency [47]. This is especially important when using EHR high-dimensional datasets like our pregnancy dataset (>27,000 variables) because having a large number of variables may lead to overfitting. In addition, having too many variables can be hard to interpret, especially when variables are correlated with each other. Moreover, the training of the PredictPTB model requires less computational time than RETAIN.

Compared to published work on preterm birth prediction, our approach has a number of advantages. First, to the best of our knowledge, this work is one of the first to consider such a large number of patients (222,436 deliveries), collected from more than 300 healthcare centers in the U.S. This large dataset enabled our model to learn for diverse patient conditions and be able to capture common as well as relatively uncommon risk factors (examples are available in Appendix Figures 13 and 14). Second, given the unbalanced classification problem where preterm deliveries are much less than full-term deliveries, our model has a high predictive performance with an ROC-AUC of 82.2*%* and PR-AUC of 40.4*%* using data available up to one month before delivery. Previous work on predicting preterm reported poor to moderate predictive performance with an ROC-AUC ranging from 59% to 74% [17, 48, 49]. Third, compared to previous work, our model is capable of leveraging the sequential and temporal trajectory of events recorded in EHR, which included large amount of information embedded in each patient’s record.

A recent work [25], which predicts preterm birth using gradient boosted decision trees on EHR data, have achieved an a ROC-AUC of 0.75 and PR-AUC of 0.40 at 28 weeks of gestation (which is approximately two months before delivery). Compared to PredictPTB, this model provides predictions starting from two months before delivery and up to 10 days before delivery, and no information was provided about the model’s performance at earlier stages of the pregnancy timeline. In addition, this work is limited to diagnosis data and doesn’t consider other data modalities. On the other hand, PredictPTB combines four data modalities (diagnosis, medications, lab orders, and procedures). This combination enables PredictPTB to learn a better representation that can capture a patient’s EHR in as much detail as possible.

Furthermore, our interpretability visualization highlighted several known risk factors for preterm birth, which establishes further confidence in our approach.

Our PredictPTB model supports the idea of personalized clinical decision support, by deriving relative importance of an individual medical code based on the context of the entire EHR history of a patient. A possible clinical application scenario would use our model to scan the medical history of an expecting mother, compute the risk score for preterm birth, and provide a visualization for healthcare providers to help them identify patients at high-risk of preterm delivery and arrange early follow-ups that could prevent complications and additional burden for the healthcare system and patients.

**Fig. 10:**
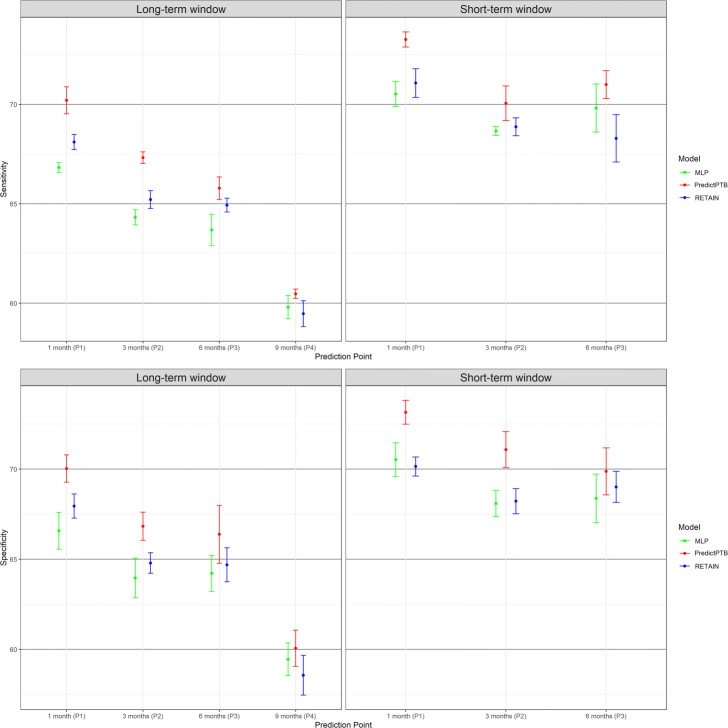
Predictive performance of the implemented models across the four prediction points using all data modalities for long-term and short-term data windows

## Conclusion

We have developed and evaluated a well-performing deep learning model for preterm birth prediction, using code-level attention-based recurrent neural networks.Our work demonstrated that temporal deep learning models can predict preterm delivery up to nine months earlier than its occurrence, using routinely collected data in electronic health records. In addition, predictions of our model are complemented by explanations that directly highlight evidence in the patient’s EHR timeline. Future work may utilize our model to provide patient-specific predictions and interpretations for more pregnancy complications (e.g. hypertension, gestational diabetes, preeclampsia, infections, and iron-deficiency anemia) and pregnancy outcomes (e.g. mode of delivery, stillbirth, miscarriage, and neonatal death).

## Appendix

### Additional results

**Fig. 11:**
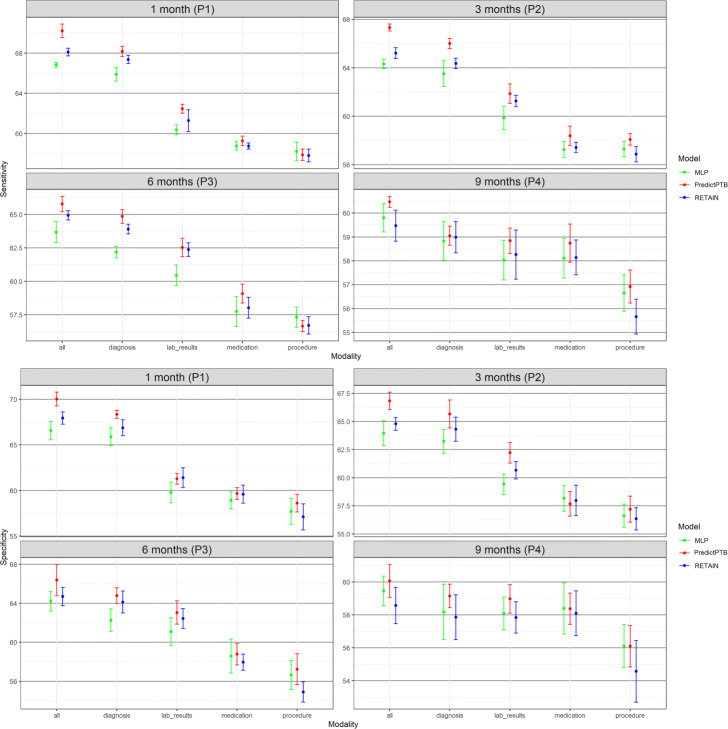
Predictive performance of models trained on single modality and combined modalities of data, across the four prediction points using long-term data window (sensitivity and specificity)

**Fig. 12:**
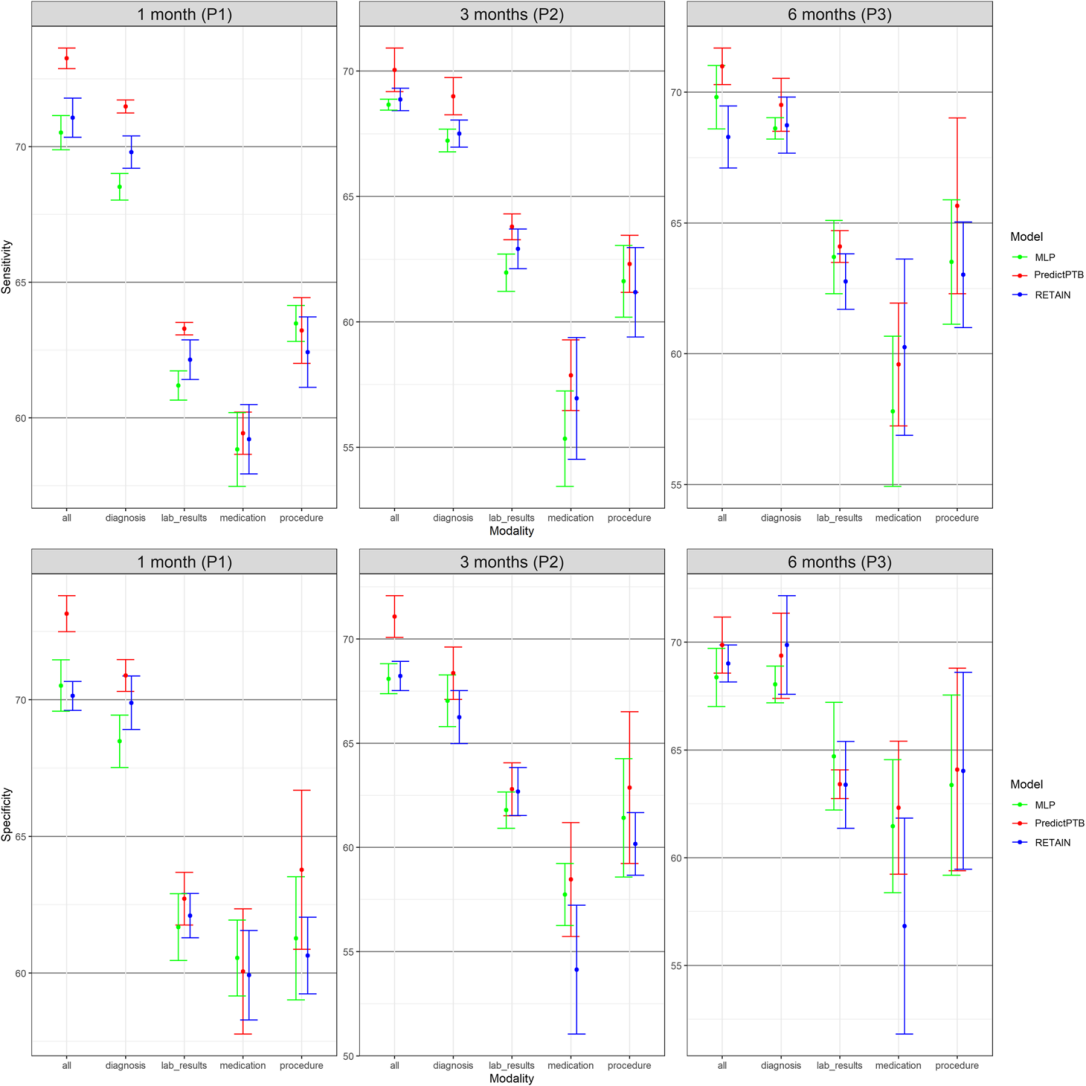
Predictive performance of models trained on single modality and integrated modalities of data, across the three prediction points using short-term data window (sensitivity and specificity)

In this section, we show more results for PredictPTB performance using sensitivity and specificity metrics. Figure 10 compares PredictPTB to MLP and RETAIN across the four prediction points, using all data modalities, for long-term and short-term data windows. Figures 11 and 12 compare models trained on single modality and combined modalities of data for long-term and short-term data windows, respectively.

**Fig. 13:**
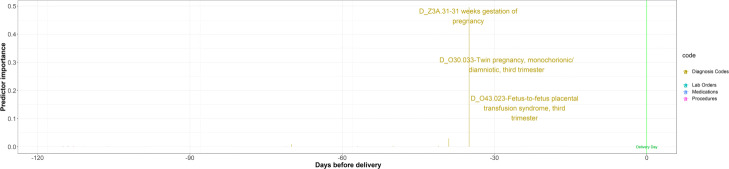
Example for a patient where PredictPTB was able to learn rare complications as risk factors for preterm birth. This patient was diagnosed with twin-to-twin transfusion syndrome (TTTS), a rare disorder which affects 10−15*%* of monochorionic, diamniotic twin pregnancies [50]. This observation is in line with literature reporting that pregnancies with TTTS complication are at increased risk for PTB [51]

**Fig. 14:**
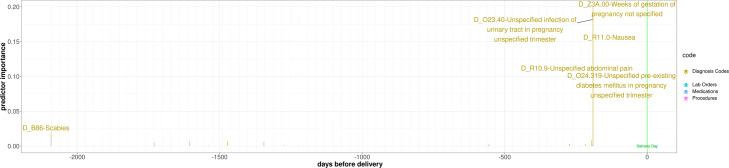
Example for a patient where PredictPTB captures common risk factors and assigns a high importance score to the visit in which these codes are documented. The highlighted visit has two important risk factors: infection of urinary tract in pregnancy and pre-existing diabetes mellitus in pregnancy

## Data Availability

The Cerner Health Facts Database (currently referred to as the Cerner Real World Data) is not publicly available. It is available to research affiliates at contributing hospitals, upon a request made directly to Cerner Corporation.

## Abbreviations

PTB: Preterm Birth
RETAIN: REverse Time AttentIoN
RNNs: Recurrent Neural Networks
ROC: Receiver Operating Characteristic
ROC-AUC: Area Under the ROC Curve
PR-AUC: Area Under the Precision-Recall Curve
